# The role of particle size of particulate nano-zinc oxide wood preservatives on termite mortality and leach resistance

**DOI:** 10.1186/1556-276X-6-427

**Published:** 2011-06-15

**Authors:** Carol A Clausen, S Nami Kartal, Rachel A Arango, Frederick Green

**Affiliations:** 1 U.S. Forest Service, Forest Products Laboratory, Madison, WI 53726, USA; 2 Department of Forest Biology and Wood Protection Technology, Forestry Faculty, Istanbul University, Istanbul, Turkey

## Abstract

Historically most residential wood preservatives were aqueous soluble metal formulations, but recently metals ground to submicron size and dispersed in water to give particulate formulations have gained importance. In this study, the specific role nano-zinc oxide (ZnO) particle size and leach resistance plays in termite mortality resulting from exposure to particulate ZnO-treated wood was investigated. Southern yellow pine (SYP) sapwood impregnated with three concentrations of two particle sizes (30 and 70 nm) of ZnO were compared to wood treated with soluble zinc sulphate (ZnSO_4_) preservative for leach resistance and termite resistance. Less than four percent leached from the particulate nano-ZnO-treated specimens, while 13 to 25% of the zinc sulphate leached from the soluble treated wood. Nano-ZnO was essentially non-leachable from wood treated with 5% formulation for the 30-nm particle size. In a no-choice laboratory test, eastern subterranean termites (*Reticulitermes flavipes*) consumed less than 10% of the leached nano-ZnO-treated wood with 93 to 100% mortality in all treatment concentrations. In contrast, termites consumed 10 to 12% of the leached ZnSO_4_-treated wood, but with lower mortality: 29% in the 1% treatment group and less than 10% (5 and 8%, respectively) in the group of wood blocks treated with 2.5 and 5.0% ZnSO_4_. We conclude that termites were repelled from consuming wood treated with nano-ZnO, but when consumed it was more toxic to eastern subterranean termites than wood treated with the soluble metal oxide formulation. There were no differences in leaching or termite mortality between the two particle sizes of nano-ZnO.

## Introduction

Zinc oxide (ZnO) has a long history of use in numerous applications in forest products and related industries, including UV stabilization in wood coatings, as an antifouling agent in marine paints, pigment and mold inhibitor in latex paints and in coatings for paper. Eco-toxicity tests from the PAN Pesticide Database state that ZnO is moderately toxic to amphibians and fish, and highly toxic to certain types of zooplankton [1]. ZnO occurs naturally as the mineral zincite and is available commercially as either micron or submicron-sized milled powders or as pyrolyzed nano-sphere dispersions. Zinc is a key component of the wood preservative ammoniacal copper zinc arsenate (ACZA), and many wood-plastic composites are treated with zinc borate [2,3].

Nano-materials often exhibit novel physiochemical properties that differ significantly from larger particles of the same material, such as their interaction with prokaryotic and eukaryotic systems [4-6]. Indeed, there have been reports of marked antibacterial activity demonstrated by nano-particles of ZnO [5,7,8]. Reddy et al. [5] report selective toxicity of nano-materials, including metal oxides, to prokaryotes and eukaryotes. Studies on eukaryotes, primarily involving mammalian cells, indicate that nano-particles of ZnO cause higher levels of oxidative stress resulting in inflammation and cytotoxicity. In prokaryotes, oxidative stress can induce cell death due to interactions between reactive oxygen species and proteins, DNA or the cell membrane [9,10]. In a previous study, our observations showed that unleached wood impregnated with particulate 30 nm ZnO caused moderate termite mortality in laboratory no-choice bioassays compared to no mortality for termites exposed to wood treated with soluble zinc sulphate (ZnSO_4_) solution [11,12].

Another possible mode of action is physical. Alexander et al. [13] showed that the mode of action for inert dust insecticides was independent of chemical reactivity. We have previously shown that concrobium dust causes mortality in *Reticulitermes flavipes *[14]. Also, inert dusts induced death in grain weevils by desiccation since respiration and ingestion did not seem to harm the insects. While ZnO is not inert, it is entirely possible that termite mortality demonstrated by Kartal et al. [12] and Clausen et al. [11,15] is the result of a physical reaction alone, or in addition to a physiochemical reaction since a film of nano-particles remained on the surface of the unleached wood blocks. Furthermore, antibacterial activity has been reported to increase with reduction in particle size [8]. In our previous study, wood blocks were treated with a single particle size (30 nm) of ZnO and the effect of particle size could not be ascertained.

The objectives of this study were twofold: to evaluate the affect of leaching on termite resistance in southern pine treated with nano-ZnO and soluble ZnSO_4 _and to evaluate the affect of particle size on termite mortality.

## Materials and methods

### Treatment chemicals

Nano-ZnO (Nanophase Technologies Corporation, Romeoville, IL, USA) was provided as an aqueous dispersion containing 50% ZnO particles (30 or 70 nm) with a proprietary dispersant. Zinc sulphate was obtained from Mallinckrodt Chemicals, St. Louis, MO, USA.

### Specimen treatment

Test specimens (25 × 25 × 6 mm), prepared from sapwood portions of defect-free southern yellow pine (SYP) sapwood were vacuum-impregnated with nano-ZnO or soluble ZnSO_4 _treatments according to American Wood Protection Association (AWPA) E10-08 standard method [16]. Pre-weighed specimens that were conditioned to 20°C and 65% relative humidity (RH) were vacuum-treated (45 min vacuum at 172 kPa) with aqueous solutions of 30 or 70 nm ZnO diluted in deionized (DI) water to 1.0, 2.5 and 5.0% based on the metal oxide (ZnO) of the dispersion (Nanophase Technologies, Inc., Romeoville, IL). Aqueous zinc sulphate solutions were prepared to contain an equivalent amount of Zn for comparison with each concentration of nano-ZnO. Untreated specimens impregnated with DI water served as controls. Treated specimens were weighed, dried at 40°C for 3 days, and re-conditioned for 2 weeks. Some treated specimens were ground to pass through a 30-mesh screen and analyzed for zinc (Zn) with inductively coupled plasma atomic emission spectrometry (ICP-AES) (Ultima ICP-AES instrument, Jobin Yvon, Inc., Edison, NJ) according to the AWPA standard method A21-00 for analysis of wood and wood treating solutions to determine the initial chemical retention in the treated blocks [17] (Table 1).

**Table 1:** Average chemical retention of pre-leached wood blocks

Treatment	Concentration	Retention (kg/m^3^)	Std dev.
Untreated	-	26.8	1.8
30 nm ZnO	1.0	45.7	1.9
30 nm ZnO	2.5	59.4	3.0
30 nm ZnO	5.0	109.1	33.7
70 nm ZnO	1.0	43.0	25.4
70 nm ZnO	2.5	67.8	25.9
70 nm ZnO	5.0	64.0	25.2
ZnSO_4_	1.0	36.9	7.4
ZnSO_4_	2.5	45.8	6.9
ZnSO_4_	5.0	74.5	9.3

### Chemical leaching

Leaching procedures were a modification of AWPA E11-06 [18]. Five specimens per treatment were placed in a 500-mL beaker and submerged in 100 mL of DI water for 14 days without agitation. The leachate water was changed after 6 h, and 1, 2, 4, 6, 8, 10, 12 and 14 days and leachate samples were collected at each time point. Leachates were analyzed for Zn with ICP-AES and the total quantity of zinc leached over the course of leaching was calculated. Percent leaching was calculated by comparing the average leach rate of five specimens per treatment to the subset of treated unleached specimens that were assayed for zinc based on initial chemical retention (Table 1).

### Termite bioassay

A no-choice termite resistance test with *Reticulitermes flavipes *Kollar (eastern subterranean termites) was performed using five leached test specimens (25 × 25 × 6 mm) for each treatment group. Termites were collected from Janesville, WI, USA. One specimen was placed in the bottom of an acrylic cylindrical container (90 mm diameter and 60 mm height) with 1 g of *R. flavipes *and moist sand. The containers were maintained at 27°C and 85% RH for 4 weeks based on AWPA E1-09 standard method [19]. Containers were periodically checked for moisture and mortality. At the end of the test, wood specimens were oven-dried, reconditioned at 27°C and 70% RH, and reweighed to calculate mass losses. Each block was visually rated using the following system: 10-sound; 9-slight attack with up to 3% cross-sectional attack; 7-moderate to severe attack with 10 to 30% of the cross-sectional area affected; 4-very severe attack with 50 to 75% cross-sectional area affected; 0-failure. Termite mortality was estimated visually.

## Results and discussion

### Leach test

**Figure 1:**
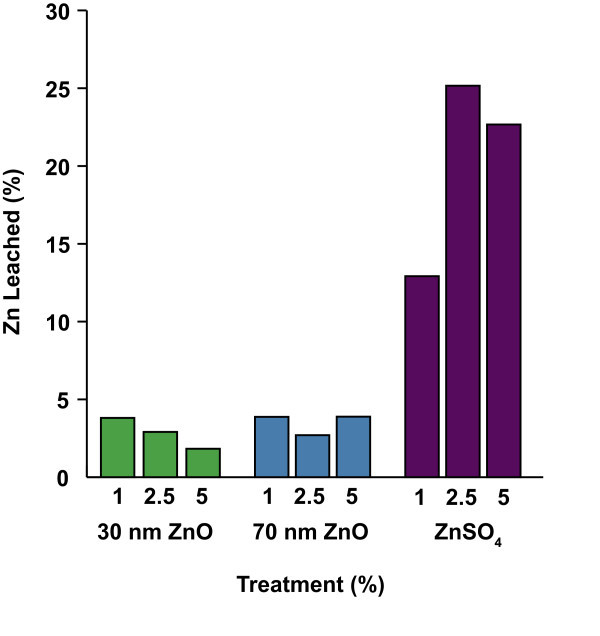
**Percent total Zn leached during 14-day laboratory leach test **[18].

Results of the leach test are summarized in Figure 1. Percent of nano-ZnO that leached was relatively low; only 1.8 to 3.9% leached from all nano-ZnO treatment groups regardless of particle size. In contrast, wood treated with a zinc sulphate solution leached to a greater degree at higher treatment concentrations. For example, 2.5 and 5.0% treatments leached 25 and 22% of the metal oxide, respectively.

### Particle size

Two sizes of nano-ZnO particles were evaluated to ascertain differences in bioactivity based on previous reports that bioactivity increases with decreasing nano-particle size [8]. In this study, no significant differences were seen between the two particle sizes tested for leach resistance, wood consumption by eastern subterranean termites or termite mortality. However, both sizes of nano-particles tested cause higher termite mortality than the soluble ZnSO_4_.

### Termite resistance

**Figure 2:**
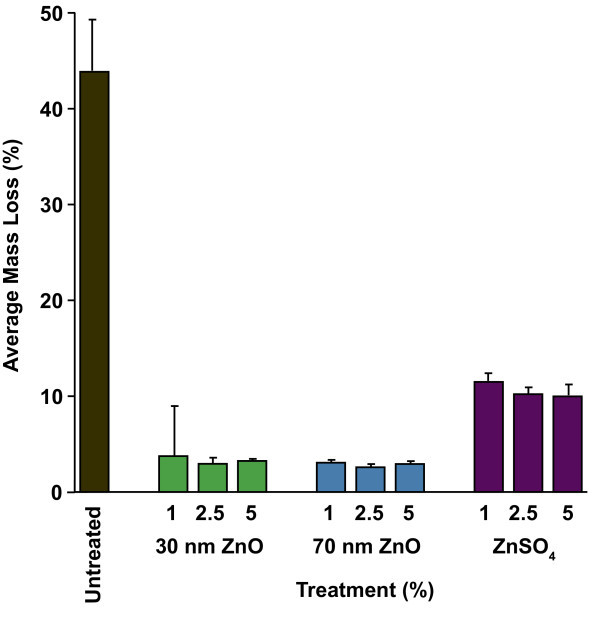
**Inhibition of eastern subterranean termite feeding by treatment of southern pine with particulate nano-ZnO and soluble ZnSO_4 _**[19].

Termite resistance results are summarized in Table 2 and Figure 2. Termite consumption of the treated wood blocks was based on visual ratings and mass loss following a standardized termite bioassay and mortality is based on visual inspection. For the nano-ZnO particulate treatments, including all concentrations and both particle sizes, wood consumption was less than 4% which is indicative of treatment non-pallitability or repellency. Despite low consumption, all nano-ZnO treatment concentrations for both particle sizes caused 94 to 99% mortality after 25 to 27 days incubation. In contrast, termites consumed slightly more (10 to 12%) of the blocks treated with ZnSO_4_, however, termite mortality was considerably lower for all three treatment concentrations. Twenty-nine percent of the termites died following exposure to 1% ZnSO_4 _but only 5 and 8% of the termites died following exposure to blocks treated with 2.5 and 5.0% ZnSO_4_, respectively.

**Table 2:** Average visual rating of damage to wood blocks by subterranean termites and estimated termite mortality

Treatment	Concentration	Ave. visual rating	Ave. mortality (%)
Untreated	-	0	12
30 nm ZnO	1.0	9	95
30 nm ZnO	2.5	9	97
30 nm ZnO	5.0	9	97
70 nm ZnO	1.0	9	94
70 nm ZnO	2.5	9	95
70 nm ZnO	5.0	9	99
ZnSO_4_	1.0	7	29
ZnSO_4_	2.5	7	5
ZnSO_4_	5.0	7	8

Higher termite mortality in wood treated with particulate nano-ZnO compared to soluble ZnSO_4 _may be due to differences in bioactivity that results from changes in the chemical structure as has been the case in other studies [20]. The pyrolyzation process alters the surface charge of nanoparticles and essentially increases the effective surface area of the metal in an evenly dispersed layer. If the particle size is smaller than the diameter of the wood window pit (< 10,000 nm) or the opening of the bordered pit (400 to 600 nm), complete penetration and uniform distribution would be expected [21].

Termiticides can generally be characterized as (1) slow-acting, non-repellent stomach poison (e.g. naphthaloylhydroxylamine, sulfuramid) [22], (2) contact or cuticular toxin (e.g. fipronil) or (3) chitin synthetase inhibitor (e.g. hexaflumuron) that mimics insect hormones which regulate a wide array of physiological functions. Some examples include interfering with molting, pupal emergence or body wall formation. Little et al. [23] reported 100% mortality to *R. flavipes *using the antioxidants butylated hydroxytoluene (BHT), flavanone and propyl-gallate as surrogates for heartwood extractives. Fipronil is known to work by blocking the gamma-aminobutyric acid (GABA)-regulated chloride channel in neurons, thus disrupting the activity of the insect's central nervous system (i.e. neurotoxin) [24,25]. Our results suggest that nano-ZnO behaves as a slow-acting, non-repellent stomach poison.

## Conclusions

Nano-ZnO (30 and 70 nm) was essentially non-leachable from wood impregnated with up to 5% of these treatments. There was no difference in leachability or termite resistance between the two sizes of nano-particles evaluated. Consumption of wood blocks treated with nano-ZnO by eastern subterranean termites was uniformly low (less than 4%) and mortality was uniformly high (greater than 94%) for all treatment concentrations tested.

## Abbreviations

ACZA: ammoniacal copper zinc arsenate; AWPA: American Wood Protection Association; BHT: butylated hydroxytoluene; DI: deionized; GABA: gamma-aminobutyric acid; RH: relative humidity; ICP-AES: inductively coupled plasma atomic emission spectrometry; SYP: Southern yellow pine; Zn: zinc; ZnO: zinc oxide; ZnSO_4_: zinc sulphate.

## Competing interests

The authors declare that they have no competing interests.

## Authors' contributions

CC conceived of the study, coordinated the research, and treated test specimens, NK performed the chemical leach testing, RA and FG conducted the termite bioassay and analysis. All authors wrote the final manuscript.
