# Correction: The role of particle size of particulate nano-zinc oxide wood preservatives on termite mortality and leach resistance

**DOI:** 10.1186/1556-276X-6-465

**Published:** 2011-07-22

**Authors:** Carol A Clausen, Nami S Kartal, Rachel A Arango, Frederick Green

**Affiliations:** 1 U.S. Forest Service, Forest Products Laboratory, Madison, WI 53726, USA; 2 Department of Forest Biology and Wood Protection Technology, Forestry Faculty, Istanbul University, Istanbul, Turkey

## Correction

Chemical retention calculations have been corrected in Table 1[1].

**Table 1:** Average chemical retention of pre-leached wood blocks.

Treatment	Concentration (%)	Retention (kg/m^3^)	**Std dev**.
Untreated	-	-	-
30 nm ZnO	1.0	8.9	0.3
30 nm ZnO	2.5	22.8	0.8
30 nm ZnO	5.0	44.3	5.2
70 nm ZnO	1.0	8.4	1.1
70 nm ZnO	2.5	21.7	2.7
70 nm ZnO	5.0	46.5	5.7
Zn SO_4_	1.0	8.7	0.4
Zn SO_4_	2.5	23.1	0.7
Zn SO_4_	5.0	43.3	1.0
